# Task-dependent estimability index to assess the quality of cardiac computed tomography angiography for quantifying coronary stenosis

**DOI:** 10.1117/1.JMI.8.1.013501

**Published:** 2021-01-09

**Authors:** Ehsan Samei, Taylor Richards, William P. Segars, Melissa A. Daubert, Alex Ivanov, Geoffrey D. Rubin, Pamela S. Douglas, Udo Hoffmann

**Affiliations:** a Carl E Ravin Advanced Imaging Labs, Department of Radiology, Durham, North Carolina, United States; b Duke University Medical Center, Department of Medicine, Durham, North Carolina, United States; c Massachusetts General Hospital, Department of Radiology, Boston, Massachusetts, United States; d Duke University Medical Center, Department of Radiology, Durham, North Carolina, United States

**Keywords:** computed tomography angiography, cardiac computed tomography, coronary vessel motion, stenosis estimation, stenosis quantification, model observer, estimability, detectability

## Abstract

**Purpose:** Quantifying stenosis in cardiac computed tomography angiography (CTA) images remains a difficult task, as image noise and cardiac motion can degrade image quality and distort underlying anatomic information. The purpose of this study was to develop a computational framework to objectively assess the precision of quantifying coronary stenosis in cardiac CTA.

**Approach:** The framework used models of coronary vessels and plaques, asymmetric motion point spread functions, CT image blur (task-based modulation transfer functions) and noise (noise-power spectrums), and an automated maximum-likelihood estimator implemented as a matched template squared-difference operator. These factors were integrated into an estimability index ($e^{\prime }$) as a task-based measure of image quality in cardiac CTA. The $e^{\prime }$ index was applied to assess how well it can to predict the quality of 132 clinical cases selected from the Prospective Multicenter Imaging Study for Evaluation of Chest Pain trial. The cases were divided into two cohorts, high quality and low quality, based on clinical scores and the concordance of clinical evaluations of cases by experienced cardiac imagers. The framework was also used to ascertain protocol factors for CTA Biomarker initiative of the Quantitative Imaging Biomarker Alliance (QIBA).

**Results:** The $e^{\prime }$ index categorized the patient datasets with an area under the curve of 0.985, an accuracy of 0.977, and an optimal $e^{\prime }$ threshold of 25.58 corresponding to a stenosis estimation precision (standard deviation) of 3.91%. Data resampling and training–test validation methods demonstrated stable classifier thresholds and receiver operating curve performance. The framework was successfully applicable to the QIBA objective.

**Conclusions:** A computational framework to objectively quantify stenosis estimation task performance was successfully implemented and was reflective of clinical results in the context of a prominent clinical trial with diverse sites, readers, scanners, acquisition protocols, and patients. It also demonstrated the potential for prospective optimization of imaging protocols toward targeted precision and measurement consistency in cardiac CT images.

## 1 Introduction

Cardiac computed tomography angiography (CTA) is a diagnostic modality for the noninvasive evaluation of coronary artery disease.^1^^,^^2^ An important CTA-derived measure is stenosis, a metric of relative geometric vessel narrowing. Stenosis is commonly calculated using manual or semiautomatically measured diameters of the diseased lumen comparing it to the normal reference lumen within each segment of the coronary vasculature containing an atherosclerotic plaque. The precision of this measurement is strongly impacted by: the extent and composition of the plaque^3^^,^^4^ (such as the presence of calcium), the patient body habitus, technical limitations that contribute to image noise and loss of spatial resolution, and image quality degradations caused by the motion of coronary vessels during image acquisition.^5^^,^^6^ Consequently, accurately and precisely estimating coronary stenosis from cardiac CTA images remains a challenging task. This challenge is evident in several multicenter clinical trials in terms of low per-patient and per-vessel specificity, inability to delineate significant stenosis, and a nontrivial percentage of nondiagnostic scans.^4^^,^^7^^–^^10^ These shortcomings are most pronounced clinically in patient cohorts with high calcium burden and high resting or labile heart rates.^4^^,^^6^

Much progress has been made in the last two decades in CTA to overcome these problems and provide a more precise and effective diagnostic scan. Many of the advancements have been focused on the technical capabilities of the CT scanners themselves. In terms of hardware, faster rotation times, dual source–detector geometries, and multisegment acquisition and reconstruction methods have been aimed at improving the effective temporal resolution of the scanner to reduce the impact of coronary vessel motion on image quality. In terms of software, metrics and algorithms have been developed to better quantify, mitigate, or correct motion artifacts at both the data acquisition and image reconstruction stages.^6^^,^^11^ In terms of patient management, pharmacologic reduction of heart rate and the use of nitrates to enlarge normal coronary segments have been used in many practices. Despite these advances, quantitative optimization of CTA in the presence of heavily calcified plaque and vessel motion remains a challenge.^12^ The current efforts to quantify the quality of the stenosis assessment do not fully account for the clinical task, the stochastic nature of cardiac motion artifacts, or the properties of nonlinear iterative reconstruction techniques used in CT today.

For a metric of image quality to add clinical utility and predictive power in the context of efficient CTA utilization and optimization, one can postulate that it must (1) be reflective of an actual clinical task as opposed to the generic aspects of image quality such as noise or blur, (2) account for the attributes of the imaging systems and techniques as they render the patient information, (3) incorporate all influencing factors into an estimator of stenosis accuracy, (4) be relatable to and validated against image quality performance as experienced clinically, and (5) be applicable to provide guidance for optimizing CTA. In line with these objectives, the purpose of this work was to develop a computational framework to objectively quantify the task performance of estimating stenosis in CTA. The proposed framework’s figure-of-merit, the estimability index ($e^{\prime }$), specifically aims to quantify the precision of an ideal estimator in delineating the true degree of coronary stenosis in CT images impacted by noise, calcium, and cardiac motion. The framework aims to provide a quantitative tool for the systematic evaluation and optimization of CTA techniques (technical or algorithmic) for stenosis assessment. In doing so, it aims to reduce the uncertainty associated with the usefulness of coronary CTA in challenging clinical cases that may be considered nondiagnostic for the characterization of a plaque or stenosis. This paper details the development of this framework, presents its validation against the results of a large clinical trial, and demonstrates its application in the context of protocol definition for coronary CT angiography.

## 2 Methods

The focus of our experimental methods was to fulfill the five objectives previously postulated as necessary for a metric of image quality to add clinical utility and predictive power to coronary CTA. In concert with those objectives, the project pursued five specific goals as listed and further detailed as follows.

(1) We first developed a library of idealized task functions, utilizing virtual coronary vessels and plaques, which incorporated the most significant elements of the designated clinical task.
(2) We mathematically modeled the attributes of the complete imaging chain, including the blurring and distortion due to cardiac motion for a variety of modern, clinically employed CT systems.
(3) A model observer framework based on the ideal maximum-likelihood estimator (MLE) was developed to quantify the stenosis estimation performance ($e^{\prime }$) as a function of patient–scanner factors.
(4) The framework was validated against a retrospective cohort of 132 patient cases from the Prospective Multicenter Imaging Study for Evaluation of Chest Pain (PROMISE)^13^ clinical trial. The framework, while devised to be genetic and applicable to any patient–scanner factor, was conditioned to reflect the span of patient attributes (anatomic, physiological, and pathological) and scanner parameters (technique, protocol, and model capabilities) in the patient cases. The developed $e^{\prime }$ metric was used as a classifier for discriminating clinically meaningful differences in image quality (as judged by expert readers and the discordance in the scores across readers).
(5) Finally, the framework was applied to ascertain the imaging parameters for optimum cardiovascular imaging as a part of the CT angiography biomarker initiative^14^ of the Radiological Society of North America (RSNA) Quantitative Imaging Biomarker Alliance (QIBA).^15^

### 2.1 Coronary Vessel and Plaque Model

To address the first requirement for developing a task-specific metric of image quality, we developed a library of idealized task functions based on the morphology explicit to our targeted indications, i.e., coronary plaques and stenoses. These functions utilize virtual coronary artery and plaque models that provide a spectrum of morphology, composition (i.e., calcium, mixed, and noncalcified), and degree of stenosis (15% to 85%) typically seen in patient populations. The task functions serve as the basis of the ideal estimator (described below) to ascertain the likelihood of assessing the degree of stenosis within a lumen.

The disease-free coronary artery was modeled as a uniform cylinder with 0.5 mm of concentric vessel wall material. The inner luminal radius was then defined according to the sex and involved segment of a given patient case with the added assumption of a right dominant coronary arterial system.^16^^,^^17^ An iodinated contrast material enhanced arterial lumen was modeled to match the contrast level measured in the ascending aorta with attenuation values adjusted to account for volume averaging expected relative to the degree of narrowing and involved vessel segment.^18^

A plaque, either noncalcified, calcified, or mixed, was included to narrow the artery. Our model assigned specific material densities and corresponding Hounsfield Unit (HU) values, according to the three typical plaque material classes: noncalcified: −40  HU, mixed: 150 HU, and calcified: 500 HU at 120 kV. Both the plaque material and the degree of narrowing were consistent with that employed in the PROMISE trial established based on consensus reads from trained clinical cardiac imagers (radiologists or cardiologists).^19^ The final parametrized vessel and plaque models, centered within a small region of interest within a uniform anatomic background (simulating epicardial fat), were then sampled at a spatial resolution at least 10× that of the targeted in-plane reconstructed voxel size. We preserved this hyper-resolution vessel and plaque model throughout the deterministic image acquisition process to better model the continuous-to-discrete image operation of modern CT imaging and to minimize potential sampling and discretization artifacts.

As coronary vessels contract and relax per heart motion, our library of idealized task functions included the temporal dimension. A given vessel segment at a given phase in the cardiac cycle may have both in-plane (axial or x−y) and through-plane (craniocaudal or z) motion vector components. However, the largest motion degradation occurs when the vessel segment is oriented perpendicular to the transverse plane and the primary motion vector lies in-plane. Therefore, our motion model was restricted to this in-plane axial motion condition. The in-plane motion direction of coronary vessel segments during healthy cardiac function is generally known and fixed.^20^^,^^21^ However, the relative direction of this motion with respect to the angular position of the x-ray source during image acquisition is random, and unless the CT source rotation time is synchronized with the cardiac motion period, the relative motion direction is uniformly random.^6^ We, therefore, modeled the relative motion direction for repeated image acquisitions as a stochastic random process with a uniform probability distribution.

Given the brief duration of cardiac half-scan acquisition periods relative to the duration of the cardiac cycle, we modeled both the motion direction and magnitude as constants for the duration of a single-image acquisition. In this framework, the motion magnitude is calculated as a function of the patient sex, resting heart rate (bpm), and the involved vessel segment with the added assumption that the phase of acquisition corresponds to the period of relative least motion (quiescence or diastasis) for the given patient-vessel combination.^20^^,^^21^ All modern cardiac CT scanners utilize patient-specific electrocardiogram information and advanced commercial algorithms to target this phase.

### 2.2 CT Imaging Operators

To address the second requirement, in terms of modeling the deterministic spatial and stochastic noise attributes of CTA, we incorporated empirically measured task-based modulation transfer functions (TTF) and noise-power spectrums (NPS) from commercial CT systems. “Task-based” refers to the measurements being made relevant to a specific plaque material (noncalcified, mixed, and calcified) targeted task with corresponding local contrast, noise, and patient attenuation size. Task-based measures of resolution and noise are a necessary component of modeling nonlinear reconstruction effects and local spectral degradations like beam hardening and calcium blooming present in cardiac CTA images.^22^

The TTF and NPS, specific to the applied scanner model, reconstruction kernel, plaque material, tube-potential (kV), and radiation dose from a wide array of clinical scanners, were applied to our idealized hyper-resolution vessel and plaque models to “image” them according to the associated imaging conditions. The deployed NPS and TTF quantities had been previously measured from images of the American College of Radiology and Mercury Phantoms scanned on machines within our clinical operation using a method described by Solomon et al.^23^ We note that while TTF can change as a function of contrast, we applied only the empirically measured TTF that best matched the primary contrast edge of a given case’s plaque material and lumen enhancement.

We included the image-domain asymmetric motion point spread functions (mPSF) induced by the complex interaction of the scanner temporal resolution, half-scan (180 deg) projection data, and coronary vessel motion vectors. Coronary vessel motion during CT acquisition results in asymmetric blurring of the vessel region within the reconstructed image due to spatial inconsistencies in the acquired projection data. Although this vessel motion is negligible in the source-to-detector direction (motion directly toward or away from the detector results in negligible magnification differences), motion in the detector channel direction (perpendicular to the source-to-detector direction) is significant relative to the detector channel size and does result in spatial inconsistencies in the projection data and subsequent asymmetric blurring. Furthermore, due to the acquisition geometry of modern third generation CT scanners, the channel direction is constantly changing as the x-ray source rotates about the object in a uniform circular motion. For a clockwise source rotation as seen from an object at the center of the scanner field of view, the channel direction can be described by the vector s=[sin(θ),−cos(θ)] where θ is the source projection angle.

To form a single instance of a two-dimensional (2D) image-domain asymmetric mPSF, the vessel motion vector of known magnitude and direction was projected onto the channel direction vector for every projection angle used to reconstruct the image. The mPSF was then calculated as the channel direction displacement path weighted by the respective projection angle reconstruction weights and rotation time of the specific CT scanner model. For dual source acquisition geometries and multisegment acquisition techniques, the mPSF was weighted and summed according to each subset of projection angles and their effective temporal resolution. This process was stochastically repeated for different uniformly random relative vessel motion directions and projection angles to form a set of mPSF instances. With each mPSF instance resulting in a unique degree of image degradation and visualized vessel deformation, the complete set of all possible mPSFs stochastically represents the mathematical imaging operator for the motion blur.

**Fig. 1:**
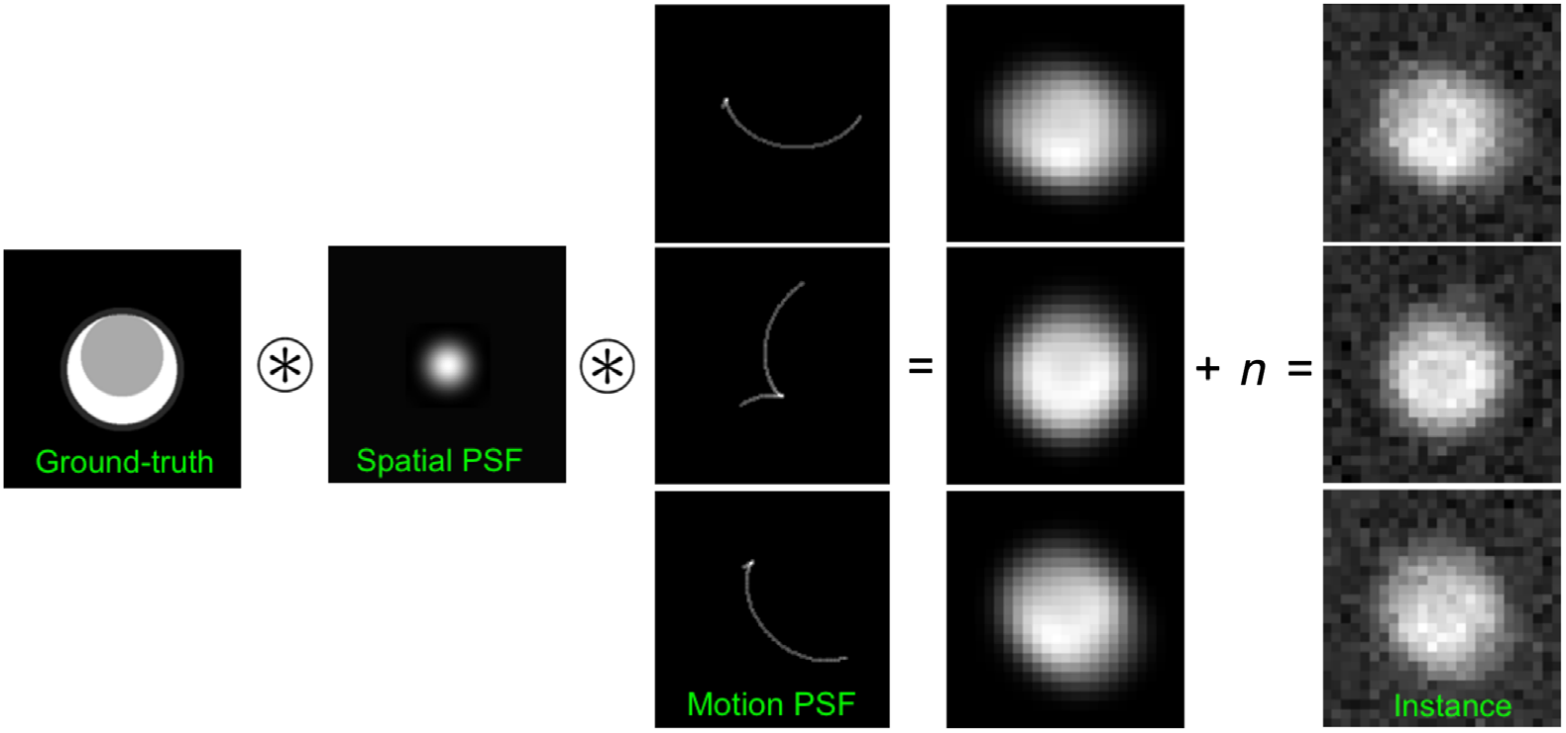
Depiction of the deterministic and stochastic CT imaging chain components that result in three example image instances. The hyper-resolution idealized ground-truth coronary plaque model is illustrated on the left representing an idealized plaque (gray) and stenotic lumen (white). Lumen example shown is 9-mm-in. diameter. First, this model is degraded by the system spatial blur (through the spatial PSF), then by instances of motion blurs (through the motion PSF, three examples shown), and finally, unique instances of correlated noise (n) are added to generate the final image instances.

Finally, to include the stochastic noise properties of the simulated imaging chain, the filtered image was downsampled to the target clinical resolution and correlated noise was added by filtering a zero-mean white Gaussian noise field (n) with the measured NPS spectrum (Fig. 1). Apart from the noise texture added through the NPS, the noise magnitude was matched to the measured noise magnitude in each case being modeled.

### 2.3 Ideal Estimation (MLE)

An estimation operator derived from statistical decision theory was deployed to address the need for an automated computational observer model. This ideal observer, based on MLE without prewhitening, considers ascertaining the degree of stenosis as its primary task and aims to minimize the squared pixel-wise difference between a noise-contaminated image instance and a noise-free template image of a known parameter value. Employing this operator on an ensemble of stochastic image instances results in a distribution of MLE estimates whose statistical dispersion or spread is reflective of the task-specific estimation performance. An estimability index $e^{\prime }$ can thus be defined as the inverse of the width of this ensemble distribution. This automated objective and ideal methodology can isolate the inherent image quality information content within the image data from estimation performance variability or degradation attributable to human observers.

Statistical decision theory dictates that for parameter estimation in the presence of Gaussian distributed stochastic noise, the ideal estimation strategy, resulting in the MLE, is to minimize the squared difference between the given noise contaminated data instance and a noise-free template data of a known parameter value.^24^^,^^25^ By finding the least squared difference with a noise-free template image of known parameter value, the ideal estimate is the same parameter value used to create the template image. This minimization process, repeated on an ensemble of noise contaminated data instances, results in a normal distribution of parameter estimates centered at the true parameter value (unbiased) with its standard deviation serving as a scalar metric of parameter specific estimation performance. The inverse of the standard deviation of this normal distribution is what we define as the estimability index ($e^{\prime }$). The estimated parameter, stenosis, is a ratio quantity between 0 and 1 (inclusive). Therefore, the inverse of the standard deviation of a distribution of estimated stenosis values can have an infinite range [0∞), but practically speaking, it is usually confined between 3 and 120. The estimability index provides an upper bound on achievable estimation performance (precision) given the characteristics of the estimated parameter task, the deterministic imaging process, and the stochastic image noise properties.

In cardiac CTA, there exist a few important caveats to this estimation model. First, because the deterministic blurring in CT results in a null space (albeit small), there may exist multiple parameter values for a given object that achieve the same minimum squared difference. The ideal estimate may correspond to multiple template values. Second, because the vessel motion in cardiac CTA results in stochastic asymmetric motion degradation, the residuals for each squared difference operation may not be “white,” meaning there may exist spatial correlations within the residuals that do not satisfy the requirement for Gaussian distributed residuals. Although these caveats do partially disqualify this operator from setting the upper bound on achievable estimation performance, it still provides a representative measure of estimation performance under realistic operating conditions. Taking these limitations into account, the ensemble of ideal estimates may include both bias and variability with respect to the ground truth value and may not be well described by the normal distribution.

**Fig. 2:**
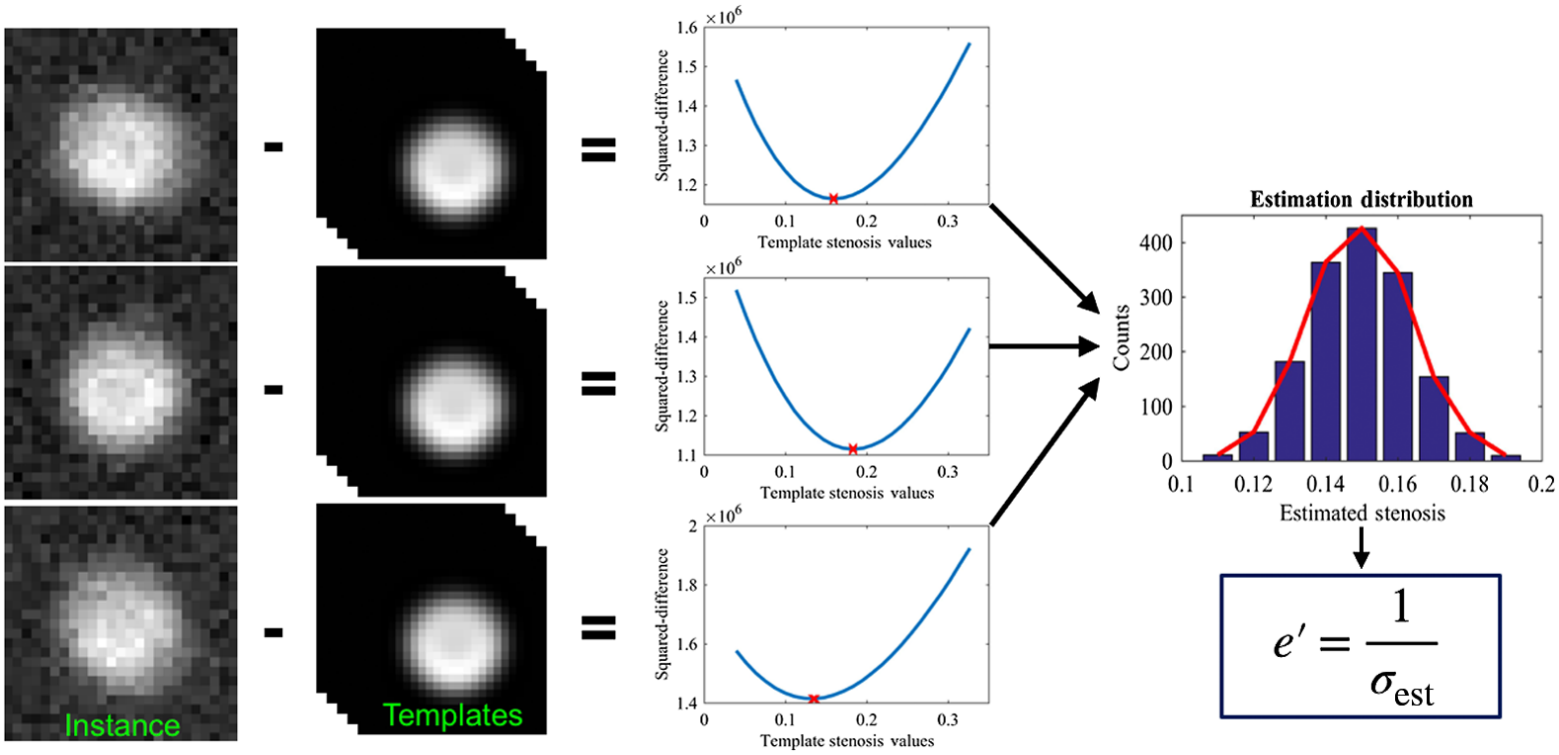
Three examples of the squared difference minimization process, which results in an ensemble distribution of estimates used to calculate the estimability index ($e^{\prime }$) for a given patient case. The stenosis value is the ratio of the diameter of the stenosed vessel to that of a normal healthy vessel. This unit, along with its percentage counterpart, is the standard for reporting stenosis in CTA.

The ideal estimator assumed that a given patient–scanner-acquisition case, as imaged, may be stochastically represented by a specific noise and motion condition. 2300 such stochastic instances of noise and motion were synthesized for a case being modeled. Each instance was then individually compared to a bank of precomputed template images to find the “ideal estimate” (i.e., pixel-wise least squared difference) for that instance. The templates provided a systematic representation of influencing factors including vessel diameter (1.5 to 5.0 mm), stenosis (15% to 85%), vessel velocity (15 to 35  mm/s), plaque material (noncalcified, mixed, and calcified), and iodinated lumen contrast (300 to 600 HU). The resultant parameter estimate distribution across the 2300 instances was then used to calculate the ideal estimator (the estimability index) for the given patient–scanner-acquisition case (Fig. 2).

### 2.4 Clinical Trial Cases Benchmark

The estimability framework was validated against a clinical dataset. The dataset was selected from the cardiac CTA arm of the PROMISE^13^^,^^26^^,^^27^ clinical trial. As there is no gold standard to ascertain the true quality of a CTA for stenosis quantification, we used clinical surrogates, the clinical image quality score, and core-site read discordance, to put clinical cases in one of two categories of high or low quality: As part of the PROMISE clinical trial, CTA cases were assessed by experienced clinical site readers for the presence and extent of stenotic plaque. These same cases were then reread by a small group of expert core lab readers, who also assigned a general image quality score (4-unit scale, with 4 being considered nondiagnostic) to each case. Relying on this data, we hypothesis that the cases that were scored high quality by core lab readers (i.e., 1) and further yielded high agreement (i.e., >50%) between the core and site readers on the classifications of stenosis are likely high-quality images. In contrast, the cases that resulted in low-quality scores by core lab readers (i.e., 3) and further yielded low agreement (<50%) between the core and site readers are likely low-quality images. No intermediate quality scores (2) were included in either cohort.

Searching across hundreds of datasets within PROMISE, 132 cases (88 high quality and 44 low quality) were identified to fall within these two divergent cohorts. The associated patient and scanner characteristics from this cohort are tabulated in Tables 1 and 2. Using this data, we test the $e^{\prime }$ estimates ability to predict this classification of cases.

**Table 1:** General patient, image, and scanner characteristics.

	High quality	Low quality	Combined
Sex^a^	52 F/33 M (3)	22 F/17 M (5)	74 F/50 M (8)
Heart rate (bpm)	64±9	69±11	65±9
Body mass index (BMI) ($\mathrm{kg}/m^{2}$)	30±6	28±5	34±7
Plaque material^b^	63 H/9 N/15 M/1 C	6 H/3 N/ 13 M/22 C	69 H/12 N/28 M/23 C
Stenosis severity (%)	33.6±16	38.3±10.5	35.2±14.6
Contrast (HU)	578±154	230±192	462±234
Noise (σ)	32±11	41±16	35±13
CNR (HU/σ)	20±7	7±8	15±10
Effective temporal resolution (ms)	280±99	274±106	278±101

a No sex reported.

b No plaque reported (H), noncalcified (N), mixed (M), and calcified (C).

**Table 2:** CT scanner manufacturer and model information.

Manufacturer	Siemens (57)	GE (44)	Philips (16)	Toshiba (15)
Model(s)	Definition (20)	LightSpeed VCT (40)	Brilliance 64 (11)	Aquilion 64 (10)
	Definition flash (13)	Discovery CT750 HD (3)	iCT 256 (5)	Aquillion ONE (5)
	Sensation 64 (13)	Discovery STE (1)		
	Sensation cardiac (5)			
	Definition AS (4)			
	Definition AS+ (1)			
Dual-source or multisegment acquisition(s)	67% (38)	2% (1)	6% (1)	60% (9)

(n) denotes the total number of patient scans (both high and low quality) represented in each category.

For each case, $e^{\prime }$ was calculated in a case-specific manner. Inputs for the estimation task included patient sex, mean, and variance of heart rate (bpm) at time of image acquisition, involved vessel [American Hospital Association (AHA) 17-segment model], stenosis severity (%), stenosis material (noncalcified, mixed, and calcified), and local luminal enhancement (HU). Inputs for the imaging attributes included manufacturer and model specific CT scanner geometry and operation (single or dual source, scan range, and multisegment optionality), rotation time (ms), reconstruction kernel and algorithm, reconstructed in-plane pixel size, slice thickness (mm), slice interval (mm), and local noise magnitude (σ).

In calculating $e^{\prime }$ for each case, the lesion with the highest percentage stenosis was selected as representative of the clinical decision. In cases where the site and core readers agreed on the highest percentage stenosis, the only remaining ambiguity resulted from multiple lesions matching the agreed upon stenosis level. In this situation, the most physiological significant or largest diameter vessel segment (assumed right dominant coronary arterial system) was selected. In cases where the site and core readers disagreed on the highest percentage stenosis, their measurements were averaged. Once determined, the involved segment was then used to calculate the coronary motion according to our protocol described in the last paragraph of Sec. 2.1.

For a small subset of patients–scanner-protocol combinations (in total, 2.5%, 200/8,000, of input parameters), there was missing or conflicting information, which was replaced by concurrent values from similar patient or scanner attributes, case averages, and published scanner details. For patients with multiple image series with differing acquisition and reconstruction parameters, the single-image series or protocol with the highest $e^{\prime }$ was selected as representative of the interpreted clinical image read.

The receiver operating curve (ROC) and its associated summary metrics were calculated for the task of classifying the high-quality versus low-quality classes for the 132 PROMISE datasets. Furthermore, we employed both a fourfold cross-validation approach and a Monte Carlo bootstrapping method using within-class case replacement, which maintained the original dataset size and class ratio to calculate confidence intervals (CI) on the class separation threshold and ROC performance.

### 2.5 Application to Protocol Optimization

An estimability index for coronary stenosis can have multiple applications including characterization and comparison of coronary imaging hardware and software, harmonization of data across clinical practices, and optimization of the imaging protocols toward targeted precision or consistency. As a demonstration, we implemented the metrology to ascertain the imaging parameters for optimum cardiovascular imaging as a part of the CT Angiography Biomarker initiative^14^ of the RSNA Quantitative Imaging Biomarker Alliance (QIBA).^15^ This implementation targeted CT factors, along with their clinically relevant ranges and levels that directly influence CTA image quality, including vessel diameter (1.5 to 5.0 mm), stenosis (30% to 70%), vessel velocity (15 to 35  mm/s), plaque material (noncalcified, mixed, and calcified), iodinated lumen contrast (300 to 600 HU), reconstructed in-plane pixel size (0.35 to 0.55 mm), noise magnitude (15 to 45 HU), scanner rotation time (0.25 to 0.35 s), dual-source acquisition (off, on), and spatial resolution (0.32 to $0.52\;{\mathrm{mm}}^{-1}$ ${\mathrm{MTF}}_{f50}$). In a full factorial design, the $e^{\prime }$ values were computed for all possible factor combinations and the results fit within a linear model to extract the associated intercept, linear, interaction, and squared terms. The results were then used to establish the combination of factors that result in the highest $e^{\prime }$.

## 3 Results

**Fig. 3:**
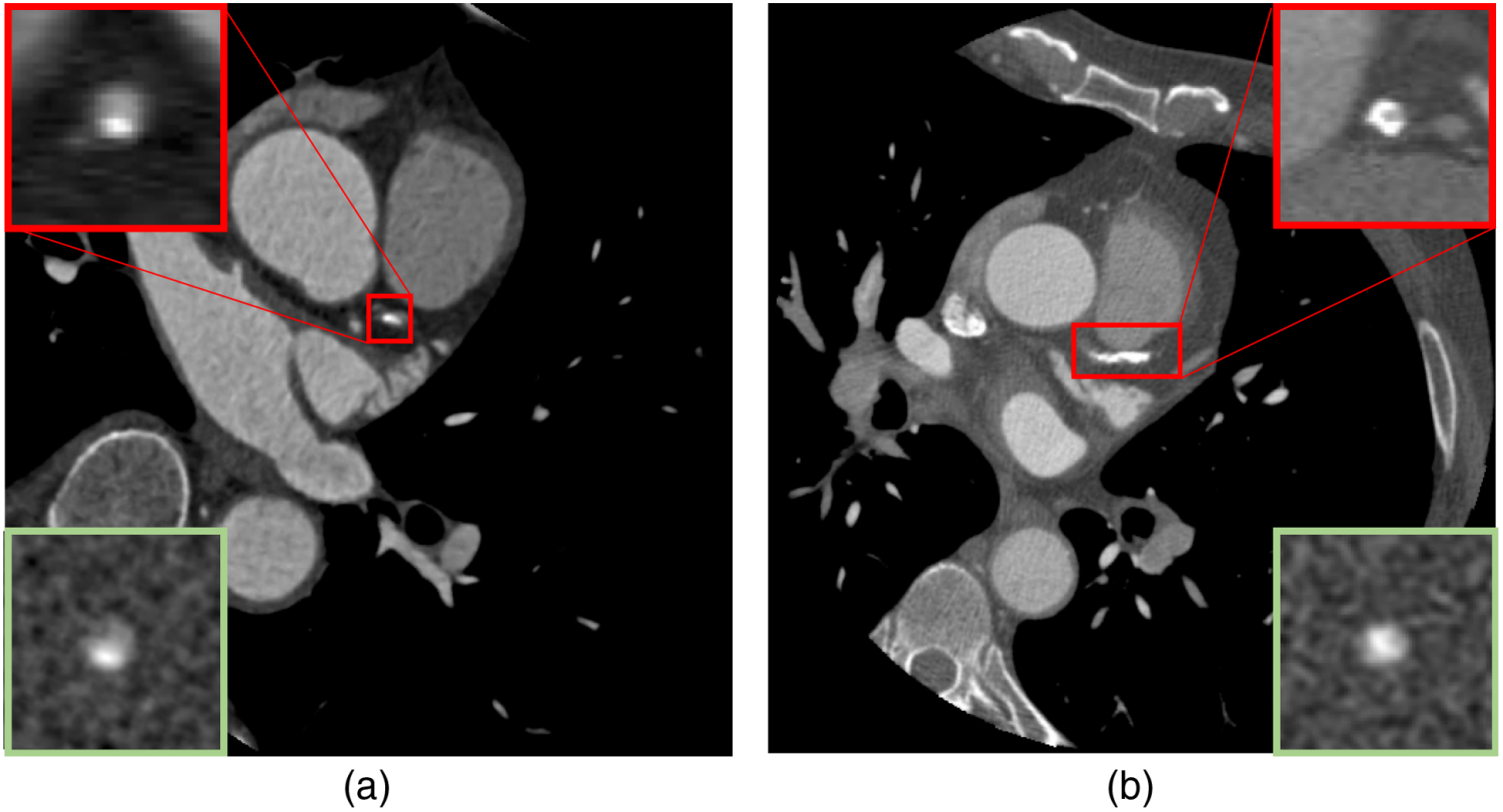
Representative examples of (a) high-quality (60% stenosis, 1.92-mm-diameter lumen) and (b) low-quality (50% stenosis, 1.85-mm diameter lumen) images from the PROMISE dataset. The magnified cross section of each case is shown at the level of stenosis as well as a close representation of the case used for the $e^{\prime }$ calculation.

**Fig. 4:**
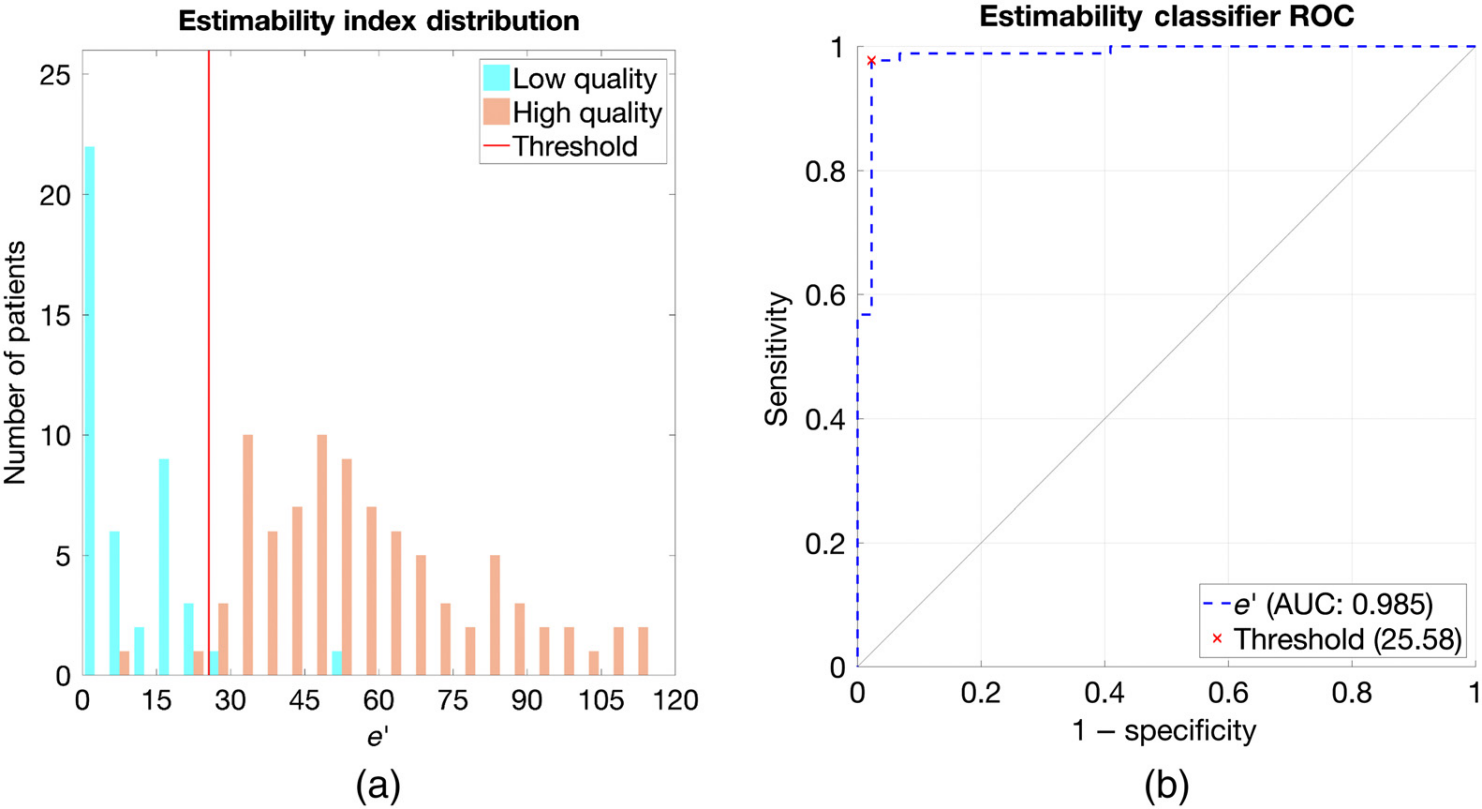
(a) The distribution of the respective classes with respect to $e^{\prime }$. (b) The ROC curve for the classification of low- versus high-image quality in the 132 PROMISE cases as a function of $e^{\prime }$.

The estimability index ($e^{\prime }$) developed in this work proved to be effective to characterize the performance of CTA in quantifying stenosis. Figure 3 offers example cases from high-quality and low-quality cases in the PROMISE dataset and their modeled analogues. The metric classified the original 132 patient datasets with an AUC of 0.985 (Fig. 4). The threshold which minimized the distance to the ideal classifier (0,1) was an $e^{\prime }$ of 25.58, which is equivalent to a stenosis estimation precision [standard deviation (SD] of 3.91%. The $e^{\prime }$ metric correctly classified 129 patient cases (accuracy of 0.977) and misclassified only three patient cases (one false-positive and two false-negatives). Close examination of these cases indicated that they either had very high or very low contrast-to-noise ratio (CNR) due to the imaging condition. The bootstrapping with case replacement and fourfold cross-validation methods resulted in similar ROC performance values for $e^{\prime }$ when compared with the original patient dataset (Table 3). These respective data resampling and training-test validation methods yielded stable $e^{\prime }$ classifier thresholds and ROC performance. These results can be considered as an indicator of the generalizability of the method.

**Table 3:** ROC performance comparison for $e^{\prime }$ correctly predicting the quality of patient cases to render high-quality concurrent classification of stenosis.

$e^{\prime }$	Original class data	Bootstrap resampling	Fourfold cross validation
AUC	0.985	0.985±0.011	0.986±0.019
Accuracy	0.977	0.979±0.012	0.966±0.030
Threshold	25.58	25.40±01.29	25.63±0.78

We found our estimability index ($e^{\prime }$) was primarily impacted by image noise, plaque composition, local luminal enhancement, vessel diameter, vessel motion (heart rate), and scanner temporal resolution (rotation time). Given the significant influence of these enumerated factors, we also found that our framework did not produce consistent results at extreme factor values for two principle parameters (local plaque CNR≤1 or vessel segment velocity ≥40  mm/s). Although there were some factor combinations that overcame this shortcoming (e.g., large slow-moving vessels on a dual-source scanner), if the CNR was too low or the velocity was too high, the other factors’ influence on image quality and parameter estimation performance was neither impactful nor visibly discernible. Practically speaking, any patient with parameter values in this range would be expected to result in nondiagnostic images for the purpose of stenosis evaluation, and consequently the $e^{\prime }$ value is expected to be zero.

**Fig. 5:**
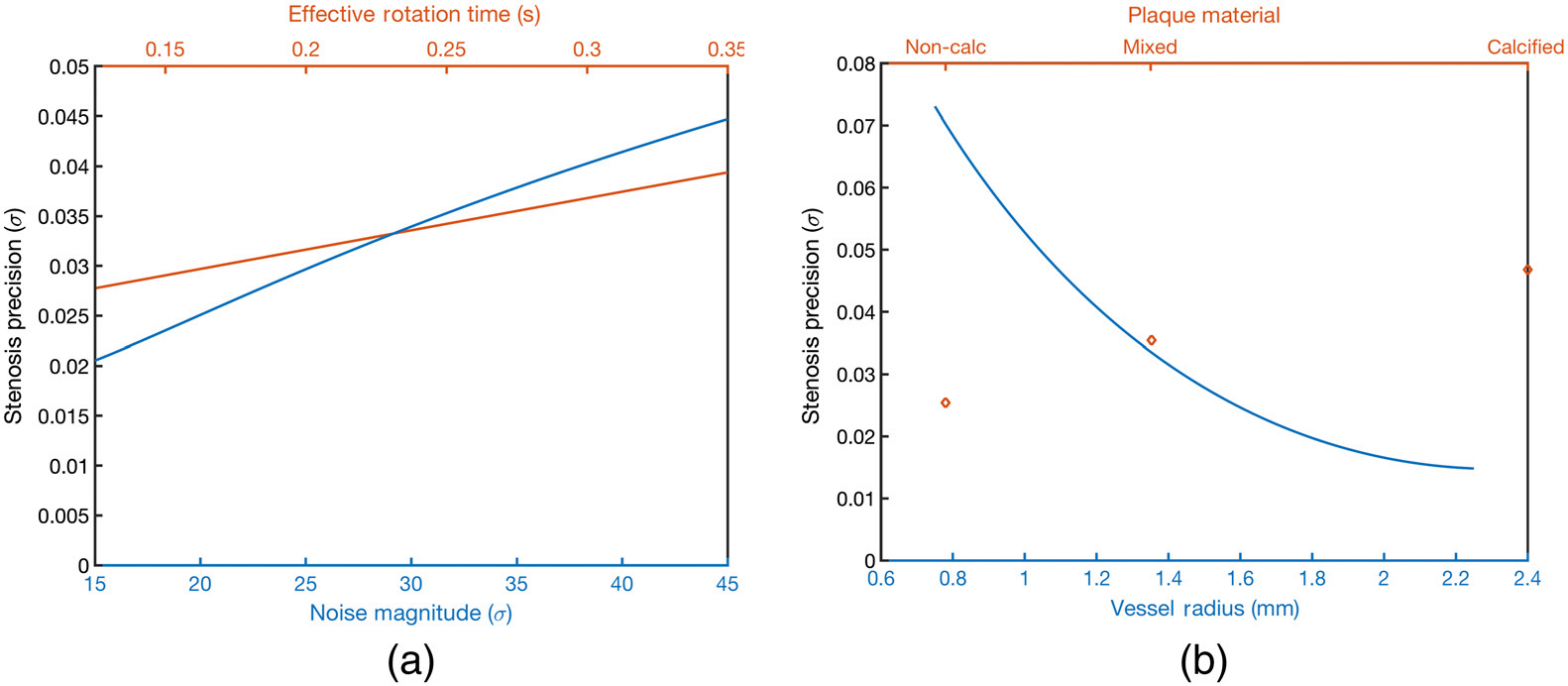
Partial dependence plots showing the relationships between factors and their average model responses marginalized across all other variables. (a) The protocol centric factors that most strongly influence the stenosis estimation performance (precision). (b) The patient centric factors that most strongly influence the stenosis estimation performance (a lower stenosis precision value denotes a higher $e^{\prime }$ value).

The analysis and fitting of $e^{\prime }$ values across the parameter space investigated for QIBA indicated that under typical or average hardware platforms and protocols choices (average response marginalized over other factors), the patient centric parameters that yielded the greatest influence on $e^{\prime }$ were vessel size (involved vessel segment reference diameter), the interaction of plaque material composition (calcium having the greatest negative impact), and luminal contrast level (Fig. 5).

**Fig. 6:**
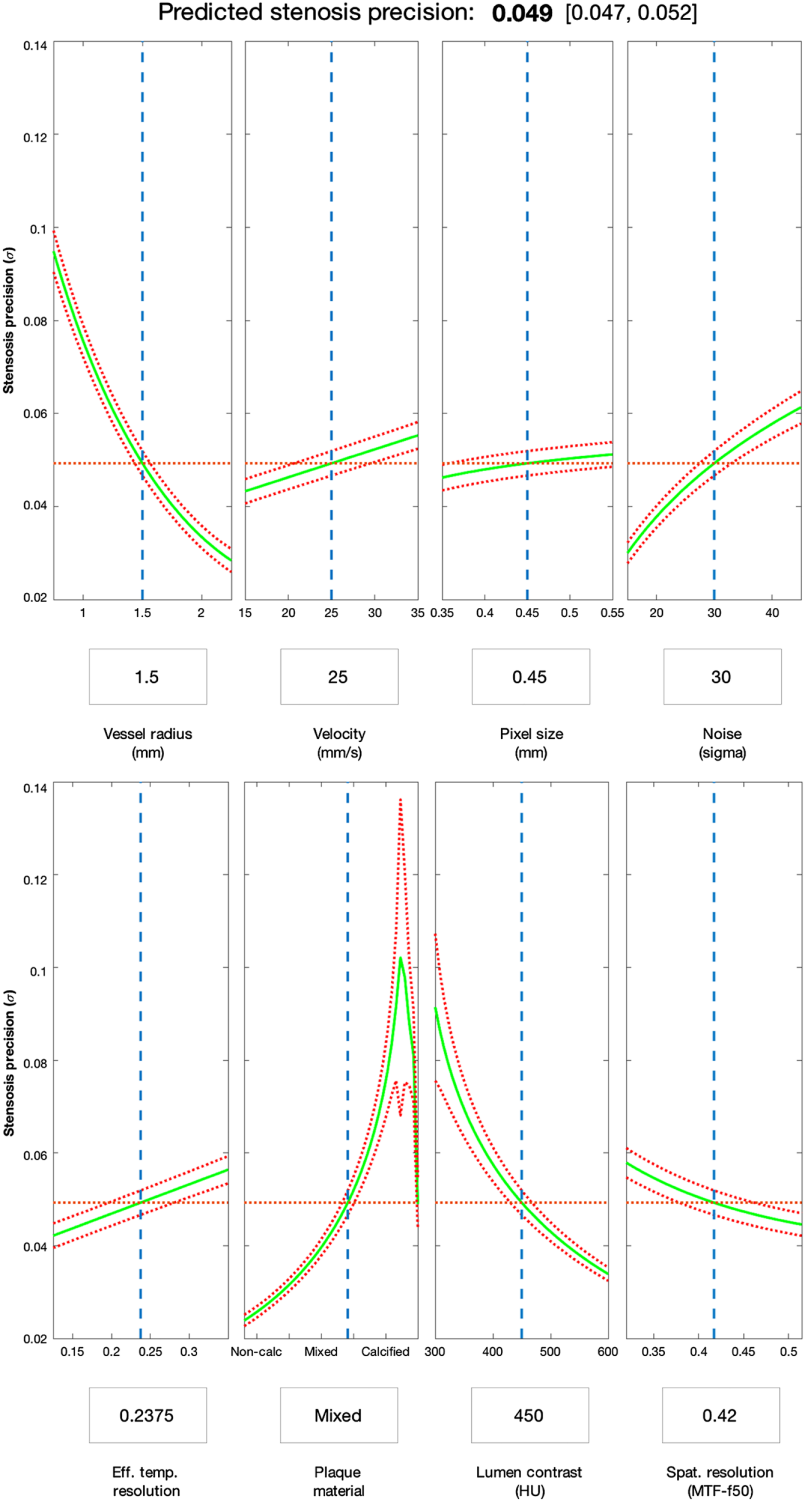
Predictive slice plots through fitted regression surface. This factor-combination demonstrates the trends and quantitative impact of each predictor under a typical mixed plaque example case. The solid green line in each plot represents the predicted stenosis precision as a function of a single predictor variable (radius, velocity, etc.), with the other predictor variables held constant at levels denoted by dashed blue lines and boxed values. The red dotted lines are the 95% confidence bounds.

The hardware and protocol factors that demonstrated the greatest influence on $e^{\prime }$ under normal or average patient conditions were image noise magnitude (σ) and effective temporal resolution (rotation time modified for possibility of dual-source scanner hardware). The respective trends and factor interactions were in line with previous published works and clinical experience, with the $e^{\prime }$ methodology offering quantitation of the expected estimation precision (Fig. 6). The fitted model contained 21 terms (including categorical levels for plaque materials) from 8 predictors and their interactions. The model had a root-mean-square error of 0.01 (fractional diameter stenosis) and a $R^{2}$ of 0.84.

## 4 Discussion

Cardiac CT is on the rise. As one of the most complex imaging procedures, many technical and patient factors can contribute to its quality, managing the optimization, and consistency of which becomes a clinical priority. This motivates the definition of metrics that can be used as surrogates of diagnostic performance for quantification, calibration, and optimization of the procedure.^28^^,^^29^ The computational estimability framework developed in this work successfully fulfilled the five requirements set forth for a metric of image quality to add clinical utility and predictive power in the context of coronary CT image quality evaluation and optimization. The framework incorporates clinically informed task information and cardiac motion, relevant attributes of imaging systems including those of the leading manufacturers and models, and precision quantification based on statistical decision theory. The framework, validated against data from a high-profile clinical trial, has demonstrated utility to be used as a metric of quality in CTA to optimize the procedure and make it more precise and consistent across the diversity of technologies, patients, and practices.

Task-specific diagnostic image quality is impacted by both patient-centric factors, largely uncontrollable (heart rate, vessel size, plaque morphology, etc.), and hardware-based controllable factors (radiation dose, spatial resolution, temporal resolution, contrast enhancement, etc.). A given factor combination and its associated interdependencies with the clinical task (stenosis estimation) determines the upper-bound on achievable estimation precision. This upper-bound is applicable to both expert readers and advanced computer segmentation algorithms. The problem we aimed to address was uncertainty in the effectiveness of coronary CTA in certain clinical settings that result in a higher than usual number of nondiagnostic CT scans and subsequently an inefficient use of high-cost imaging technology. The developed task-based metric of image quality $e^{\prime }$ attempts to quantitatively account for all relevant factors in a computational framework consistent with both physics principles and statistical decision theory. Many of these factors are common to CT more generally (spatial resolution, noise, beam hardening, etc.), but motion is particularly challenging in cardiac CT due to the constant cyclical pumping of the heart. For example, the common half-scan acquisition and reconstruction approach employed in cardiac CT aims to reduce the impact of this motion by minimizing the acquisition period. However, this half-scan approach combined with high coronary vessel velocities introduces asymmetric motion artifacts and image degradations that are difficult to “read through” (i.e., so-called prewhitened) due to their stochastic nature.

In this study, we devised a framework to account for the prominent degradation factors that affect the characterization of cardiac stenosis. The innovative and necessary inclusion of analytically derived mPSFs and their treatment as a stochastic image degradation factor was critical in the proper application of ideal estimation theory in the context of cardiac CT.^15^ To our knowledge, this is the first instance of ideal estimation theory applied in the context of diameter stenosis estimation performance. The framework was capable of incorporating the primary patient and scanner factors and their interactions that influence cardiac CT into a single scalar index of image quality $e^{\prime }$. The index demonstrated excellent classification performance on a clinically relevant dataset.

Our gold standard of quality for the validation of $e^{\prime }$ was dictated by lack of absolute ground truth for the quality of CTA (i.e., it is nearly impossible to know the absolute truth of a patient, as even surgical interventions will alter the state of the vessel). We thus relied on the current clinical standard based on expert judgment. We ascertained high- and low-quality cases based on the concordance of two observer-based assessments, quality score, and concordance between readers. Cases that rendered high score and high concordance on stenosis assessment were binned as high quality and vice versa. But each assessment is imperfect. The image quality score rendered by readers globally lumped together all possible causes of degraded image quality (e.g., high noise, motion artifact, and contrast mistiming). Likewise, reader concordance is affected by variations in interpretation environments (e.g., realities of clinical practice, level of risk averseness, and experience) and limitations of inter-reader variability (κ≅0.69).^19^ Nonetheless, we believe combining these respective surrogates of image quality and using them to form bins of quality, the breath of which is reflective of the level of granularity than can be ascribed to such surrogates, overcomes these limitations. The method enabled us to select patient cases whose differences in image quality were sufficiently disparate as to cause a measurable difference in clinical interpretation. This method, in fact, enabled us to use a gold standard that in actuality results in clinically meaningful differences in estimation performance.

In this work, we validated $e^{\prime }$ against a clinical dataset. Although the application of this estimation framework with the 132 retrospectively selected clinical cases is limited, it provides encouraging initial results in the context of a large clinical trial with multiple scanners and sites. Although we did not anticipate our model to exactly predict these case outcomes, especially considering that our effective gold standard was expert reader consensus (naturally subject to inter- and intrareader bias and variability), the results are promising and offer additional insight into clinical image quality in cardiac care. For example, our $e^{\prime }$ validation study’s optimal ROC operating point threshold (25.6) provides a quantitative value for delineation between respective classes with demonstrated clinical significance. The stenosis estimation precision that separated the low quality from the high-quality patient cases was equivalent to a 15.6% estimation precision [95% CI]. For context, the most recent professional guidelines for the interpretation and reporting of CTA diameter stenosis estimates suggests the use of measurement bins approximately 25% wide.^19^ This suggests that as the effective estimation performance nears the categorization or quantization precision, readers are more likely to disagree on their diagnostic measurement. Therefore, if the clinical and research community aim to effectively triage patients for invasive angiography or further improve or refine the predictive power for clinical outcomes based on cardiac CT stenosis severity or plaque characterization, the underlying image quality must be optimized and improved to achieve a more precise stenosis measurement.

This $e^{\prime }$ framework may enable prospective image quality and diagnostic performance optimization of new or existing technologies and protocols by ascertaining patterns and interactions within the modeled factor dependencies and architecture. Our ongoing work in conjunction with the RSNA QIBA^15^ to ascertain optimum imaging conditions for quantitative tasks serves as a demonstration. Example outcomes included in this paper show that estimation precision can be systematically predicted and optimized for specific patient–protocol combinations or targeted patient cohorts. For example, for a patient with a high pretest probability of calcium, our model suggests that a relatively high dose, sharp reconstruction kernel, and fast rotation can provide a quantitative improvement in stenosis estimation. This is, of course, a sensible approach already recommended by professional societies and guidelines, but this platform provides a clinically relevant metric through which the quantitative performance can be predicted and image quality trade-offs with respect to radiation dose, contrast dose, and heart rate can be systematically understood and incorporated in the technology or optimization choice.

Practical use of the $e^{\prime }$ methodology requires certain knowledge about the patient ahead of the scan. One such patient-specific factor is the material composition and density of the coronary plaque. Our model incorporated specific material densities and corresponding HU values according to the three plaque material classes informed by clinical expectations (noncalcified: −40  HU, mixed: 150 HU, and calcified: 500 HU at 120 kV). However, one can assume certain ranges of possibilities for a given patient and prospectively plan the scan in such a way that minimizes the likelihood of an imprecise exam across a range of plaque possibilities. Further work will be beneficial to test this prospective strategy and whether the predications can prove clinically effective.

Although our developed platform does incorporate, and is sensitive to, the primary factors that influence image quality and diagnostic performance, it did not isolate the sources of image degradation separately (e.g., high noise, motion artifact, and contrast mistiming). Further, there were other factors that were not included in the model. For example, we did not explicitly include spectral effects, such as calcium blooming and local beam hardening that can negatively impact image quality and make image interpretation near impossible in high calcium burden patients. Although patients with a prescan probability of calcium or very high coronary artery calcium score on noncontrast CT may not be the candidates for stenosis evaluation through CTA, these factors are important. Likewise, our motion model focused primarily on motion within the more influential axial plane and not in the craniocaudal direction, even though the influence is likely less prominent. Future studies may explicitly include these aspects of image quality.

Finally, while our methodology systematically characterized key factors influencing CTA performance, we note that our study did not account for many clinical factors that influence the accuracy of CTA in patient care and clinical outcomes. Those include additional patient variability and cardiac conditions, testing, catheterization, and hospitalization.^13^^,^^27^ These factors were, in fact, present in the clinical dataset that was used for benchmarking $e^{\prime }$ and likely the very reason for $e^{\prime }$ not achieving a perfect prediction. This limitation was due in part to a lack of data (i.e., cases with systematic variations in these factors) as well as the essential mathematical first-principle foundations of the $e^{\prime }$ framework. The framework was, in fact, designed to provide an idealistic foundational basis to understand the influence of CTA imaging conditions, supplemented with additional layers of models that incorporate the other influencing factors. Exploring additional and wider ranges of patient, task, and imaging variabilities is a natural extension of this work.

## 5 Conclusions

A computational framework was successfully implemented to objectively estimate task performance in quantifying coronary stenosis using an ideal MLE in cardiac CT. The framework was validated against clinical results in the context of a subset of a large clinical trial (PROMISE) with diverse sites, readers, scanners, acquisition protocols, and patient characteristics. The developed task-based metric of image quality ($e^{\prime }$) proved predictive of quantifying and classifying scan quality and may be suitable for patient-centric protocol optimization in cardiac CT to maximize diagnostic image quality.
